# Pictorial essay: PET/CT in tuberculosis

**Published:** 2008-05

**Authors:** S Harkirat, SS Anana, LK Indrajit, AK Dash

**Affiliations:** Department of Nuclear Medicine and PET/CT Facility, Delhi Cantt, New Delhi - 110 010, India; Department of Radiodiagnosis and imaging, Army Hospital (Research and Referral), Delhi Cantt, New Delhi - 110 010, India

In routine practice, oncologic workup is responsible for the majority of referrals for whole-body FDG-PET/CT. The indications in oncology include staging, restaging, assessment of therapy response, and detection of recurrence. In spite of the great success achieved by FDG-PET imaging in the evaluation of malignant disorders, the modality is not specific for the diagnosis of cancer.[1] It has been noted that processes such as infection and inflammation, and particularly granulomatous diseases, also cause increased FDG uptake in the affected tissues.[2–6]

Tuberculosis (TB) is a chronic granulomatous inflammation caused by *Mycobacterium tuberculosis*. India accounts for nearly a third of the global burden of tuberculosis, with approximately 1.8 million new cases of tuberculosis reported every year.[7] Although it involves the thorax most frequently, any organ system in the body can be infected. The clinical and radiological features of tuberculosis are known to mimic those of many other diseases. The role of FDG PET and PET/CT in TB and other inflammatory diseases is evolving and is not as yet clearly defined.

At the same time, there is a considerable increase in PET/CT referrals for patients with fever of unknown origin (FUO), generalized lymph node (LN) enlargement, and mediastinal or abdominal lymphadenopathy, especially when other investigations are inconclusive. The aim of such referrals is generally to rule out an underlying malignant disease or to detect an inflammatory pathology. Infections remain the most frequent cause of FUO, followed by neoplasms and noninfectious inflammatory diseases.[8,9] In India, TB is known to be the commonest infection to present as FUO.[10] ′The high sensitivity of FDG PET in detecting malignant lesions, infections, and other inflammatory processes alike, makes it an important tool that has the potential to play a role in the diagnostic protocol and management of patients with FUO.′[11,12]

While performing FDG PET/CT for oncologic workup, we found TB to be a common cancer mimic, producing uptake patterns that are indistinguishable from that of cancer. Many studies have documented increased FDG uptake in active TB in diverse anatomical locations, mimicking malignant processes.[5,6,13–17] Though a high standardized uptake value (SUV), greater than 2.5, is attributed to malignant lesions,[18] we have encountered high values of peak SUV, upto 21.0 (range 2.2-21.0), in tuberculous lesions Figures [1–7].

**Figure 1 (A-E):**
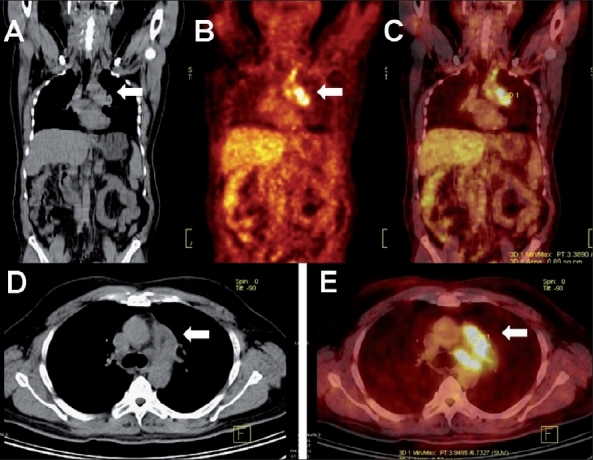
Mediastinal lymphadenopathy in a patient of carcinoma colon who, post-hemicolectomy and post-chemotherapy was detected to have raised tumor marker (CEA) levels. Coronal plain CT (A), PET (B), PET/CT (C) with axial plain CT (D), and PET/CT (E) images of the thorax show FDG-avid mediastinal nodes (SUV 6.7) (arrows). No pulmonary lesion was detected on the CT images. The patient underwent mediastinoscopy and lymph node biopsy, which revealed tuberculosis

**Figure 2 (A-C):**
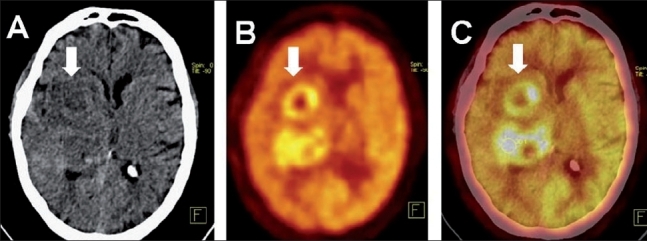
Cerebral tuberculous abscess. A 70-year-old diabetic with acute-onset hemiparesis. Axial plain CT (A), PET (B), and PET/CT (C) images show hypodense lesions in the right basal ganglia and thalamus (arrows) with a mass effect. PET shows two ′doughnut lesions,′ with peripheral FDG concentration and central cold areas. The pattern is suggestive of, though not specific for, tuberculous abscesses. Stereotactic biopsy revealed tuberculosis

**Figure 3 (A-D):**
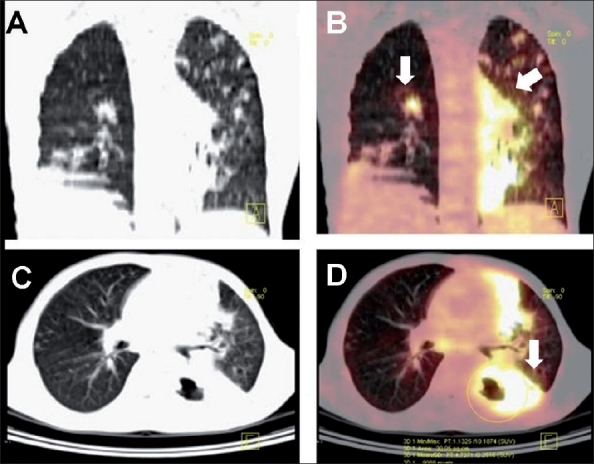
Pulmonary tuberculosis. A sputum positive case of pulmonary TB with clinically poor response to 2 months of antituberculous therapy. Coronal plain CT (A) and PET/CT (B) with axial plain CT (C) and PET/CT (D) images reveal extensive FDG-avid pulmonary parenchymal lesions. The superior segment of the left lower lobe shows consolidation with central cavitation (arrows) with an SUV_max_ of 10.1. These fi ndings suggest active disease, indicating an inadequate response to therapy

**Figure 4 (A-D):**
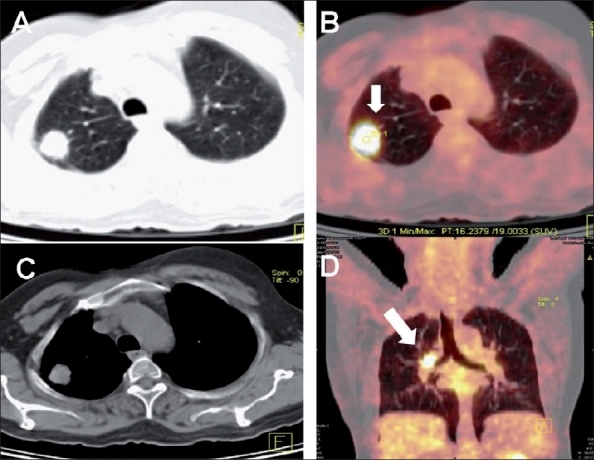
Pulmonary tuberculoma. Axial lung (A) and mediastinal window (C) CT images show a soft tissue density nodule in the right upper lobe of the lung. Axial (B) and coronal (D) PET/CT images reveal intense FDG uptake in the nodule (SUV_max_ of 19.0) and an enlarged right parabronchial node (arrows), suggesting a malignant neoplasm. Wedge resection and histopathologic examination of this solitary pulmonary nodule revealed TB. In TB endemic countries like India, FDG-avid lung lesions need to be interpreted cautiously

**Figure 5 (A-F):**
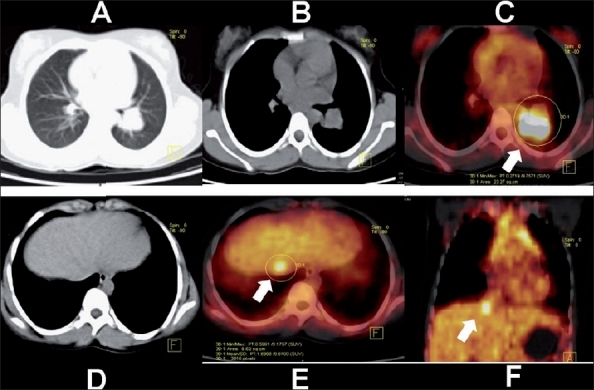
Tuberculous hilar lymphadenopathy associated with a solitary focal hepatic lesion. A chest radiograph in a 14-year-old girl with cough revealed a left-sided hilar mass. Axial CT thorax with lung (A) and mediastinal (B), axial PET/CT (C), axial abdominal CT (D) and PET/CT(E), and coronal PET/CT (F) images show intense FDG uptake in the enlarged left hilar nodes (SUV 9.7) and focal uptake in the liver, subdiaphragmmatic in location (arrows). The Mantoux test was strongly positive, along with the TB polymerase chain reaction test. The patient was started on antituberculous therapy

**Figure 6 (A-D):**
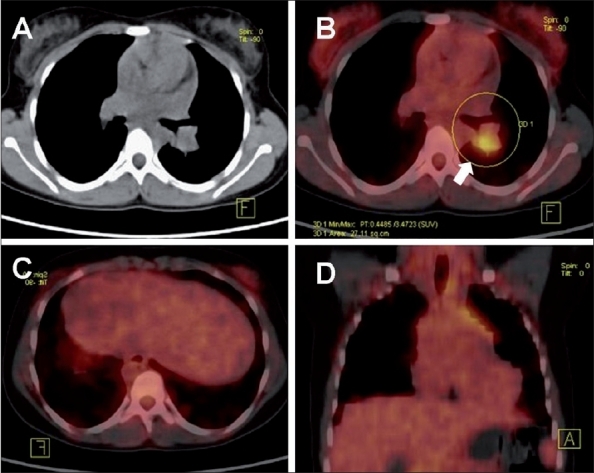
Repeat axial CT (A), PET/CT (B) images of the thorax, and axial (C) and coronal (D) PET/CT abdomen images of the same patient described in Figure 5 show complete resolution of the liver lesion and a considerable reduction in the size of the left hilar mass and the intensity of the FDG uptake (arrow) (SUV_max_ 3.4). PET was useful in assessing response to therapy

**Figure 7 (A-D):**
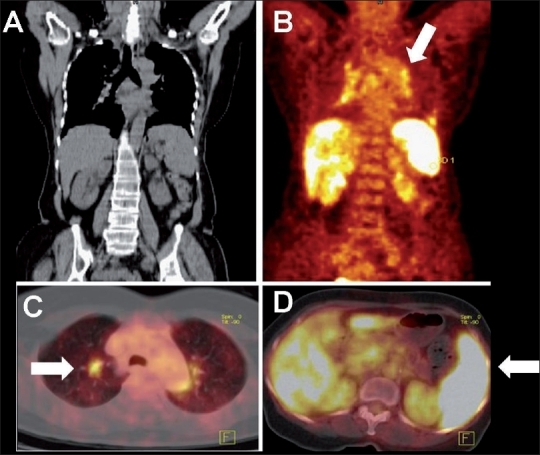
Disseminated TB with multifocal hepatic and diffuse splenic uptake. Coronal plain CT (A) and PET (B) with axial PET/CT (C, D) images in a patient with FUO show diffuse increased FDG uptake in an enlarged spleen and multifocal uptake in the liver, mediastinal nodes, and patchy lung lesions (arrows). Transbronchial sampling from the subcarinal nodes revealed tuberculosis. PET in this case guided the tissue sampling from an active lesion in an accessible site, using the least invasive route

## Case Material

All studies were performed on an integrated PET/CT scanner (Biograph 2, Siemens Medical Solutions, Erlangen, Germany). A whole-body PET/CT study, from the skull base to the mid-thigh level, was performed 1 h after intravenous injection of 370 MBq of FDG. Nonenhanced CT scan images were obtained, using 130 KV and 100 mAs. CT-based attenuation correction was done. Images were reconstructed using a standard iterative algorithm and reformatted into transaxial, coronal, and sagittal views. Fusion images of PET and CT were obtained. The PET/CT images were evaluated by one radiologist and one nuclear medicine physician in all cases. Focal accumulation of FDG above the background muscle uptake in an abnormal location was considered a positive finding. Areas of increased uptake were evaluated qualitatively and quantitatively using standard methods.[19] The peak SUV (SUV_max_) of the abnormal areas was noted.

**Figure 8 (A-C):**
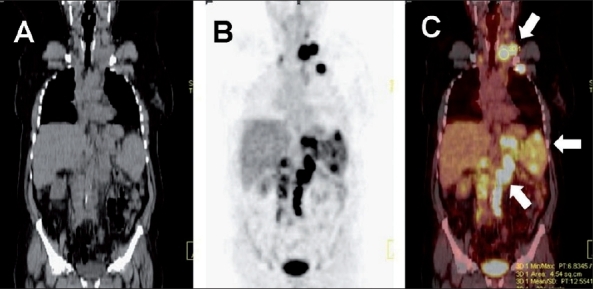
Disseminated TB with focal splenic lesions, sparing of the liver and with extensive lymph node involvement. Coronal CT (A), PET (B), and PET/CT (C) images in a patient with cervical lymphadenopathy show large FDG-avid cervical, supraclavicular, and abdominal nodes. FDG-avid focal lesions are seen in the spleen (arrows). The differential diagnoses included lymphoreticular malignancy (lymphoma), metastases from an unknown primary, and tuberculosis. A supraclavicular lymph node biopsy confi rmed tuberculosis. Note the relative sparing of the liver

**Figure 9 (A-D):**
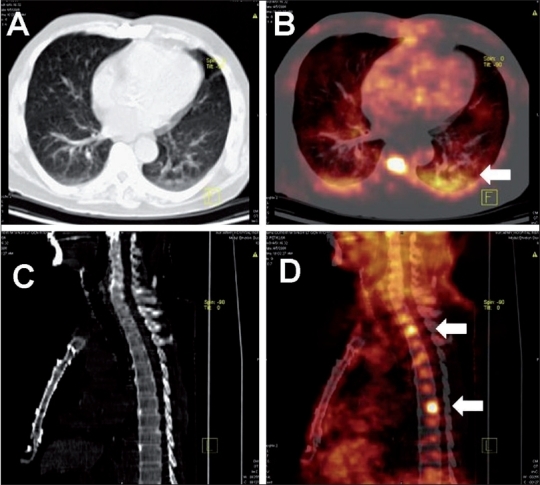
Pulmonary and spinal tuberculosis. Axial CT chest (A), axial PET/CT (B), sagittal reconstructed spine (C), and sagittal PET/ CT (D) images in a patient with FUO show FDG-avid reticulonodular lesions in the left lung with associated FDG-avid foci in two dorsal vertebrae (arrows), with no bony lesion seen in the corresponding CT images. The patient responded well to empirical antituberculous therapy

**Figure 10 (A-F):**
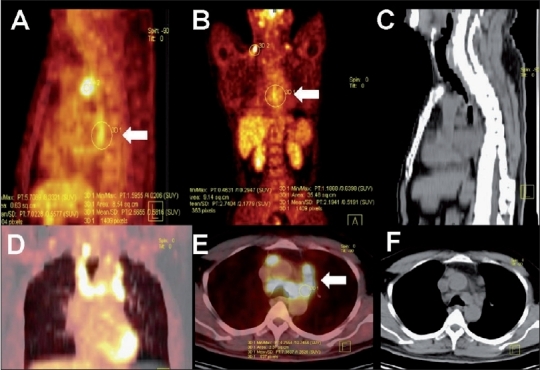
Tuberculosis of the esophagus. Endoscopy in a 42-year-old man with dysphagia revealed a mid-esophageal stricture. Sagittal (A) and coronal (B) PET, sagittal plain CT (C), coronal PET/CT (D), axial PET/CT (E), and axial CT (F) images of the thorax show increased FDG uptake in the mildly thickened walls of the mid-esophagus (arrows), with multiple FDG-avid nodes in the mediastinum and supraclavicular regions. Carcinoma of the esophagus was suspected. Biopsy from the esophagus and the supraclavicular lymph nodes revealed tuberculosis

**Figure 11 (A-E):**
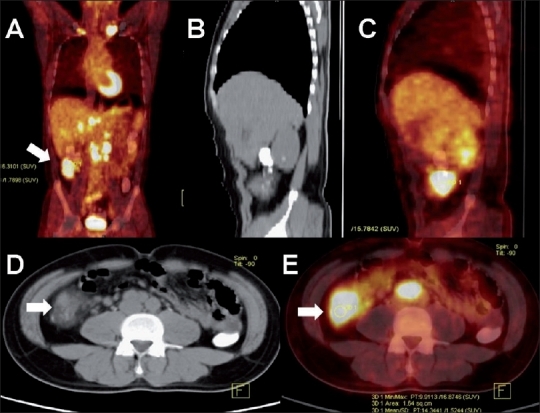
Tuberculosis of the cecum. Coronal PET/CT (A), sagittal plain CT reconstruction (B), sagittal PET/CT (C), axial plain CT (D), and axial PET/CT (E) images of the abdomen in a 36-year-old man with generalized lymphadenopathy shows increased FDG uptake in the cecum (arrows), with irregularly thickened walls (SUV 16.8) and multiple enlarged supraclavicular and abdominal lymph nodes. Disseminated carcinoma of the cecum was suspected. Colonoscopic biopsy of the cecal lesion and cervical lymph node biopsy, both revealed tuberculosis. The patient responded well to antituberculous therapy

**Figure 12 (A-F):**
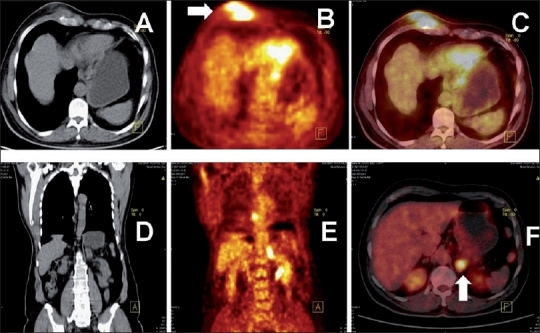
Tuberculosis of chest wall and adrenal gland. Axial (A) and coronal (D) plain CT, PET (B, E), and axial PET/CT (C, F) images in a 57-year-old man with an anterior right chest wall swelling shows intense subcutaneous FDG uptake within a soft tissue mass involving the fi fth costochondral junction, associated with increased FDG uptake in an enlarged left adrenal gland (arrows). A soft tissue chest wall sarcoma with adrenal metastasis was suspected. Biopsy of the chest wall lesion revealed TB. The lesions regressed following anti-tuberculous therapy

**Figure 13 (A-E):**
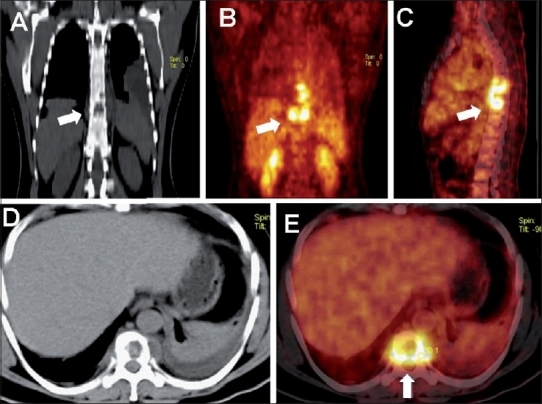
Tuberculosis of the spine. A coronal plain CT reconstruction (A) in a 42-year-old man with upper back pain shows osteolytic lesions involving multiple contiguous mid-dorsal vertebrae (arrows), with a paravertebral cold abscess and a left-sided pleural effusion, suggesting tuberculosis. Coronal (B) and sagittal (C) PET with axial plain CT (D), and corresponding PET/CT (E) images acquired to evaluate the extent of the disease show intense FDG uptake in the affected thoracic vertebrae. No other FDG-avid lesion is detected. The patient responded well to anti-tuberculous therapy

Patients with a diagnosis of tuberculosis were identified from the database. A large number of these were referred for workup of FUO or lymphadenopathy [Figures 5–9]. Others included suspected cases of malignancy that turned out to have tuberculosis [Figures 2 and 10–12] and follow-up cases of cancer found to have associated TB [Figure 1]. A few patients had established TB and were referred for evaluation of the extent of disease or to monitor response to treatment [Figures 3 and 13]. Table 1 lists all the cases described here.

**Table 1:** Case details

Fig. no	Organ System	Location of lesion on PET/CT	Indication for PET/CT	Original diagnosis	Method of confirming diagnosis	Uptake intensity (SUV_max_)	CT features
1	Lymphoreticular	Mediastinum	Ca. colon follow-up	Mediastinal nodal metastases	Mediastinoscopy and LN biopsy	7.9	Discrete 10-18 mm sized mediastinal nodes
2	Brain	Basal ganglia and thalamus (right)	Mass lesion - brain	Glioma	Stereotactic biopsy	9.4	Hypodense lesion with mass effect. No calcification.
3	Thorax	Lungs (bilateral parenchymal lesions)	To assess response to ATT	Tuberculosis	Sputum AFB (+)	10.1	Bilateral reticulonodular opacities, consolidation with cavitation
4	Thorax	Lung (SPN)	To assess nature (benign *vs* malignant)	? Carcinoma lung	Wedge resection and biopsy	19.0	Soft tissue density lung nodule with enlarged parabronchial node (right)
5,6	Lymphoreticular	Hilar adenopathy	To assess nature (benign *vs* malignant)	? Tuberculosis ? Lymphoma	Tuberculosis PCR, Mantoux, response to ATT	9.7	Hilar lymph node mass (left)
7	Multisystem	Liver, spleen, lung parenchyma, mediastinal nodes	FUO	? Tuberculosis ? Lymphoma	Transbronchial sampling of mediastinal nodes	6.1-12.4	Hepatosplenomegaly, patchy lung consolidation
8	Multisystem	Spleen, abdominal and supraclavicular nodes	FUO	? Tuberculosis ? Lymphoma	Supraclavicular lymphnode biopsy	14.3	Retroperitoneal lymphadenopathy, hypodense lesions in spleen
9	Skeletal	Dorsal spine, lung	FUO	? Tuberculosis	Response to empirical ATT	6.4	Reticulonodular lung lesions. Bones normal.
10	GIT	Esophagus, mediastinal nodes	Esophageal stricture	? Ca. Esophagus	Esophageal biopsy	3.6-10.1	Mediastinal lymphadenopathy
11	GIT	Caecum, abdominal and supraclavicular nodes	Generalized lymphadenopathy. Lump in Rt. iliac fossa	? Ca. caecum	Cecal and LN biopsy	16.3	Caecal mass, abdominal and supraclavicular nodes
12	Multisystem	Chest wall, adrenal gland	Chest wall swelling; to assess nature (benign vs malignant)	? Soft tissue sarcoma	Biopsy from the chest wall mass. Response to ATT.	8.6	Soft tissue mass fifth costochondral junction (right). Enlarged left adrenal gland.
13	Skeletal	Dorsal spine	To assess disease extent	Caries spine	Response to empirical ATT	7.8	Osteolytic lesions in mid-dorsal spine, paravertebral cold abscess, and pleural effusion

## Role of Accompanying CT Scans

CT images of PET/CT were helpful in characterizing the lesions morphologically and in some instances, especially in lung and bone lesions, were indicative of a tuberculous etiology. We did not use intravenous contrast enhancement for our CT scans. However, the use of intravenous contrast may increase the specificity of diagnosis in some instances, by more accurately demonstrating the presence of necrosis in enlarged lymph nodes or the presence of typical focal lesions in the liver and spleen.

## Organs

### Central nervous system

According to Kang *et al*., ′the possibility of a tuberculous brain abscess should be considered when FDG accumulates at the periphery of a ring-enhancing lesion in a chronically ill or immunocompromised patient.′[20] FDG-PET shows intense tracer uptake at the periphery of the lesion in a ring-like or ′doughnut′ pattern, with low uptake within the abscess cavity [Figure 2].

### Thorax

Intense FDG uptake is usually noted in active tuberculous lesions involving the lung parenchyma[6,21] [Figure 3]. This is attributed to the presence of a large number of activated macrophages which have a high glycolytic rate. The CT images in PET/CT may add useful morphological information in defining the nature of the lung lesions. PET may help in determining the activity in the lesions, define the extent of disease, and aid in assessing the response to therapy. High FDG uptake has been reported in tuberculomas[16] [Figure 4].

A common dilemma faced during oncologic workup with FDG-PET/CT is the presence of FDG-avid mediastinal or hilar nodes in cases of extrathoracic malignancies (e.g., carcinoma colon, renal cell carcinoma, or carcinoma cervix). In these entities, isolated mediastinal nodal metastases are uncommon and tuberculosis may be the cause of FDG-avid mediastinal or hilar adenopathy [Figure 1].

### Lymphoreticular system

In mediastinal, supraclavicular, and intra-abdominal tuberculous lymphadenitis, a high focal uptake of FDG has been reported.[21–23] We found FDG-avid tuberculous nodes in diverse locations [Figures 1,5,7 and 8]. Also, disseminated TB can variably involve the liver and spleen. In proven cases of disseminated TB, we found varied patterns of increased FDG uptake in the liver and spleen, some showing diffuse and others focal uptake [Figures 7 and 8].

### Skeletal system

*Osteomyelitis:*  Tuberculous osteomyelitis is a common entity in Asian countries, with frequent involvement of the spinal column. This entity classically involves contiguous dorsal or lumbar vertebral bodies and the intervening discs, often associated with abscess formation and granulation tissue. FDG-PET has a high sensitivity for the detection of chronic osteomyelitis.[24] Tuberculous lesions are found to have increased FDG uptake in the active regions of granulomatous inflammation, with cold areas that represent necrosed tissue (pus)[25] [Figures 9 and 13].

### Abdomen

Although TB can involve any part of the gastrointestinal tract, from the esophagus to the anal canal, the most commonly involved regions are the distal ileum and cecum. The lesions may be ulcerative, proliferative, or ulceroproliferative. The latter type may present as a bowel mass, indistinguishable from bowel cancer on routine imaging modalities and may exhibit intense uptake on FDG-PET imaging[14] [Figures 10 and 11]. Involvement of the adrenal gland may also present with FDG avidity [Figure 12]. The role of FDG-PET in assessing the urinary system is limited because of the interference caused by the high concentration of the excreted FDG in urine, which masks FDG-avid lesions.

### Dual time point imaging

Studies have documented the value of additional delayed images, obtained 90-120 min after FDG injection, in differentiating benign from malignant lesions. On delayed images, inflammatory lesions show increased FDG washout, whereas cancerous lesions usually exhibit further accumulation of tracer.[26,27] However, in our experience, in 15 TB patients, we found equivocal results with dual time point imaging at 45 and 120 min post-FDG injection; a majority of the tuberculous lesions showed no reduction, a few showed mild reduction (up to 20%), and many showed an increase (varying from 10-40%), in peak SUV.

### The future

FDG-PET has a high sensitivity for infection and inflammation but poor specificity. One approach that may increase the diagnostic accuracy of PET for tuberculosis includes the combined use of F-18 FDG and C-11 acetate, as the latter accumulates in tumors but not in inflammatory lesion.[28] Thus, C-11 acetate may help differentiate inflammation from neoplasms. In the future, labeling antituberculous drugs like isoniazid and rifampicin with positron emitting isotopes may culminate in the development of TB-specific PET radiopharmaceuticals.

## Conclusion

Due to the high prevalence of tuberculosis in India, false positive cases during oncologic workup with FDG-PET are commonly encountered in practice. Though FDG PET/CT is not specific for tuberculosis, it plays an important role in the evaluation of known or suspected TB cases. FDG PET/CT can determine the activity of lesions, guide biopsy from active sites, assess disease extent, detect occult distant foci, and evaluate response to therapy. Active tuberculous lesions often exhibit a high degree of FDG uptake, though this can vary, depending upon the grade of inflammatory activity. No characteristic pattern has been identified as yet that will definitely differentiate them from cancerous lesions.

With FDG-PET imaging *per se*, based on semiquantitative analysis using SUV and the dual time point imaging technique, it is currently not possible to differentiate malignant lesions from active tuberculosis consistently. However, with an integrated PET/CT technique, the CT scan images may help differentiate tuberculosis from malignant lesions, using morphologic criteria. The use of intravenous contrast increases this ability. In future, new, more specific radiotracers, like positron-emitter labeled antituberculous drug molecules may help to differentiate TB from cancer and nontuberculous inflammatory processes.
